# Whole-Genome Sequence of *Mycobacterium tuberculosis* Korean Strain KIT87190

**DOI:** 10.1128/genomeA.01103-14

**Published:** 2014-10-30

**Authors:** Young Kil Park, Heeyoon Kang, Heekyung Yoo, Seung Heon Lee, Hanseong Roh, Hee Jin Kim, Sungweon Ryoo

**Affiliations:** a Korean Institute of Tuberculosis, Osong, Chungbuk, Republic of Korea; b Macrogen, Geumcheon-gu, Seoul, Republic of Korea

## Abstract

*Mycobacterium tuberculosis* is a contagious agent that causes tuberculosis. A specific type (called the K cluster) of *M. tuberculosis* with 10 copies of IS*6110* in restriction fragment length polymorphism (RFLP) has been found in about 4% of *M. tuberculosis* isolates in Korea. Here, we report the complete genome sequence of *M. tuberculosis* Korean strain KIT87190 belonging to the K cluster.

## GENOME ANNOUNCEMENT

Tuberculosis (TB) is still a major health problem in Korea. According to a World Health Organization global report, Korea’s TB prevalence and incidence were 146 and 108 per 100,000 population in 2012 (1).

We previously reported 5 clusters among 138 *M. tuberculosis* isolates from the 7th Nationwide Tuberculosis Prevalence Survey by the IS*6110* restriction fragment length polymorphism (RFLP) typing method (2). One of the clusters was also found in outbreak cases among high school students (3) and in a local province (4) in Korea. Thereafter, the specific type of strain with 10 copies of IS*6110* was called the Korean (K) cluster, which was distributed in approximately 4% of Korean TB patients. However, we found recently that these K clusters showed different variable-number tandem repeat (VNTR) patterns (5). When we analyzed 52 *M. tuberculosis* isolates belonging to the K cluster in IS*6110* RFLP, which were isolated from different TB patients without any transmission links by Supply 24 VNTR loci (6, 7), we found that the biggest VNTR cluster included 16 isolates (30.8%). This cluster had copy numbers 5, 3, 4, 9, 4, 4, 7, 2, 3, 3, 4, 2, 4, 3, 3, 2, 4, 5, 2, 4, 2, 3, 2, and 1 in VNTR loci 2163b, 0424, 1955, 4052, 3192, 4156, 2996, 0802, 3690, 4348, 2165, 0960, 2401, 1644, 3007, 0580, 2347, 2531, 2461, 0577, 2059, 3171, 0154, and 2687, respectively. We then selected one strain (KIT87190) for whole-genome sequencing, which was susceptible in first- and second-line antituberculosis drugs.

To successfully resolve the complexity of *M. tuberculosis* KIT87190, we sequenced the genome through the shotgun sequencing method using the GS-FLX Titanium system. All 290,851 reads with 224,001,875 bases from the shotgun library were assembled into one scaffold using Newbler software version 2.8 (Roche, Germany) with 3-kb and 8-kb GS FLX paired-end libraries (130,664 and 129,673 reads, respectively). We also sequenced 49,712,246 reads with 5,020,936,846 bases (1,123× coverage) of an Illumina HiSeq 2000 paired-end library for gap closure and homopolymer error correction. The 91 gaps among the contigs were closed by GapCloser (version 1.10), GapFiller (version 1.11), and primer walking on PCR products (8).

The *M. tuberculosis* KIT87190 genome comprises one chromosome of 4,410,788 bp with a G+C content of 65.6%. In total, 4,027 genes comprising 3,911 coding sequences (CDS), 62 pseudo genes, 3 rRNA genes (5S, 16S, and 23S), 45 tRNAs, and 6 ncRNAs were annotated using NCBI Prokaryotic Genome Annotation Pipeline (PGAP; http://www.ncbi.nlm.nih.gov/genome/annotation_prok).

We confirmed that 10 copies of IS*6110* and 6 copies of IS*1081* were within the genome. Also we verified that all 33 VNTR loci (MIRU-02, MIRU-04, MIRU-10, MIRU-16, MIRU-20, MIRU-23, MIRU-24, MIRU-26, MIRU-27, MIRU-31, MIRU-39, MIRU-40, ETR-A, ETR-B, ETR-C, ETR-F, QUB15, QUB26, QUB11a, QUB11b, Mtub04, Mtub21, Mtub24, Mtub29, Mtub30, Mtub34, Mtub39, VNTR2372, VNTR3336, VNTR4156, VNTR3232, VNTR4120, and VNTR3820) were intact.

### Nucleotide sequence accession number.

The complete genome sequence of *M. tuberculosis* KIT87190 has been assigned the GenBank accession number CP007809.
